# Internal Limiting Membrane Peeling versus Nonpeeling to Prevent Epiretinal Membrane Formation following Vitrectomy for Posterior Segment Open-Globe Injury

**DOI:** 10.1155/2021/3152728

**Published:** 2021-08-28

**Authors:** Wen-Long Wei, Zhong Lin, Ming-Na Xu, Ke-Mi Feng, Zhen-Quan Zhao

**Affiliations:** ^1^The Eye Hospital, School of Ophthalmology and Optometry, Wenzhou Medical University, Wenzhou, Zhejiang, China; ^2^Department of Eye Trauma, The Eye Hospital of Wenzhou Medical University, No. 270 Xue Yuan Xi Road, Wenzhou, Zhejiang 325027, China

## Abstract

**Purpose:**

Approximately 30% of patients with an open-globe injury (OGI) develop a secondary epiretinal membrane (ERM). This study was performed to assess whether internal limiting membrane (ILM) peeling in the treatment of posterior segment OGI prevents ERM formation.

**Methods:**

The medical records of 33 patients who underwent vitrectomy for posterior segment OGI from 2016 to 2019 were retrospectively analyzed. Of these patients, 17 underwent ILM peeling during the vitrectomy and 16 did not. The patients' demographic and surgical data were collected. The associations of ILM peeling with the preoperative findings of posterior segment OGI and development of a postoperative ERM were analyzed. Student's *t*-test was used to evaluate differences in continuous variables, and the chi-squared test or Fisher's exact test was used for categorical variables. Time-to-event curves were calculated from postestimation Cox proportional hazards models.

**Results:**

An ERM developed in three eyes (17.6%) in the ILM peeling group and in eight eyes (50.0%) in the nonpeeling group (*p* < 0.05). There was no statistically significant difference between the groups in visual acuity at baseline (1.68 vs. 1.58 logMAR, *p*=0.68) or at final follow-up (0.72 vs. 0.78 logMAR, *p*=0.66). Median visual acuity significantly improved in both groups (*p* < 0.001). In the multivariable models, ILM peeling (odds ratio, 0.19; 95% confidence interval, 0.04–0.91; *p*=0.04) and worse preoperative vision (odds ratio, 0.29; 95% confidence interval, 0.10–0.80; *p*=0.02) were associated with lower likelihood of ERM formation.

**Conclusion:**

Preventative treatment with ILM peeling contributed to decreased development of an ERM in patients with OGI involving areas near the fovea.

## 1. Introduction

Open-globe injury (OGI) is a common ophthalmic disease that may cause blindness. It can lead to serious complications such as traumatic cataract, retinal detachment, and posttraumatic endophthalmitis with the potential need for enucleation [1]. The correct diagnosis and treatment are essential for the prevention of vision loss [2]. Approximately 30% of patients with an OGI develop a secondary epiretinal membrane (ERM); among patients with an intraretinal foreign body, the prevalence rises to 60% [2]. ERMs may cause macular damage, increase the number of surgeries required, and affect postoperative visual acuity (VA).

Retinal pigment epithelium (RPE) cells that are released or migrated to the vitreous can form an ERM [3]. The internal limiting membrane (ILM) can provide a scaffold on which RPE and glial cells settle and proliferate [4]. Thus, ILM peeling may help to reduce ERM formation. In the treatment of proliferative vitreoretinopathy (PVR) [5] and primary rhegmatogenous retinal detachment (RRD) [6], ILM peeling may successfully prevent the development of a postoperative ERM. However, the effect of ILM peeling on preventing an ERM after surgical treatment of OGI remains unclear.

Researchers have proposed that the risk of macular ERM formation increases as the damaged area becomes closer to the macula [7]. Previous studies have revealed the importance of removing the posterior hyaloid face in patients with macular disease [8] and the role of ILM peeling in the prevention of ERM recurrence [9]. To better manage OGI near the macula and reduce the occurrence of ERM, we retrospectively analyzed the effect of ILM peeling during pars plana vitrectomy (PPV) on the prevention of ERM in patients with OGI involving the retinal area near the fovea.

## 2. Methods

**Figure 1:**
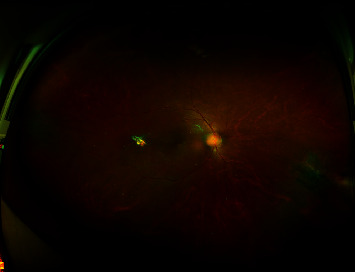
Postoperative wide-angle fundus photograph showing the lesion located 2.5 papillary diameters from the central fovea.

This study was conducted at the Eye Hospital of Wenzhou Medical University (Zhejiang, China). Informed consent was not required because of the retrospective nature of the study. We reviewed the electronic medical records of all patients diagnosed with posterior segment OGI from January 2016 to December 2019. The inclusion criteria were a diagnosis of posterior segment OGI with damage to the retina or choroid, localization of the lesion between 1 papillary diameter (PD) and 4 PD from the central fovea (Figure 1), no macular involvement in the retinal detachment, and ≥3 months of follow-up. The exclusion criteria were detection of an ERM during the initial vitrectomy performed to repair the ocular trauma, a history of any posterior segment disorder, postoperative optical coherence tomography (OCT) photographs with poor image quality that could not be used for analysis, and the presence of any maculopathy (e.g., diabetic retinopathy, idiopathic ERM, or age-related macular degeneration).

All patients underwent a 23-gauge standard three-port PPV (Stellaris PC; Bausch + Lomb, Laval, Quebec, Canada) under retrobulbar anesthesia or general anesthesia. Phacoemulsification was performed, if necessary, but no intraocular lens was implanted simultaneously. Intraocular foreign bodies (IOFBs) were removed through a separate pars plana incision. If no posterior vitreous detachment (PVD) was present, a manual PVD was performed. The retina was inspected for incarceration or direct injury, including impact by the IOFB. An argon green 532 nm endolaser was applied in one or two rows around the retinal breaks, and other procedures were performed as deemed necessary in each case. Next, 0.5 mL of 0.25% indocyanine green (Dandong Yichuang Pharmaceutical Co., Ltd., Liaoning, China) was injected over the posterior pole. ILM peeling was carefully performed in the macular area and the area around the wound with ILM forceps (725.44 Alcon Grieshaber Advanced; Alcon, Geneva, Switzerland) after removal of the excess dye. All cases included fluid-air exchange followed by sterile air or silicone oil (RT SIL-OL 5000 sterile silicone oil; Carl Zeiss Meditec AG, Jena, Germany) endotamponade. Postoperatively, patients received routine topical drops and systemic anti-inflammatory treatment.

At each postoperative visit, the patients underwent measurement of VA, slit-lamp examination, wide-angle fundus photography, and spectral-domain OCT (Heidelberg Engineering, Inc., Heidelberg, Germany). The primary outcome measure was the occurrence of ERM in the macular region at any point during the follow-up period. ERM formation was defined as the appearance of a hyperreflective line internal to the ILM of the macular region on a spectral-domain OCT scan. The secondary outcome measures were the central foveal thickness and final VA.

The patients' clinical and demographic characteristics and follow-up data were recorded and analyzed using IBM SPSS Statistics, Version 25 (IBM Corp., Armonk, NY). Student's *t*-test was used to evaluate differences in continuous variables, and the chi-squared test or Fisher's exact test was used for categorical variables. Time-to-event curves were calculated from postestimation Cox proportional hazards models. The time to event was calculated as the time from the operation to either the first occurrence of an ERM or the time of last follow-up if no ERM occurred. For the statistical analysis, VA in decimal fraction was transformed to the logarithm of the minimum angle of resolution (logMAR). Low VA states were recorded as follows: counting fingers (logMAR = 1.7), hand motion (logMAR = 1.8), light perception (logMAR = 1.9), and no light perception (logMAR = 2.0) [6].

## 3. Results

The current study included data from 33 eyes of 33 patients aged 21–53 years who met the aforementioned inclusion criteria. Of these 33 patients, 32 were male (97.0%). The preoperative baseline Snellen VA was worse than counting fingers in 29 eyes (87.9%). Corneal wounds were noted in seven eyes in the nonpeeling group and eight eyes in the ILM peeling group. Central corneal scars were found in four eyes in each group. Corneoscleral wounds were seen in one eye in the nonpeeling group and three eyes in the ILM peeling group. Scleral wounds were seen in eight eyes in the nonpeeling group and three eyes in the ILM peeling group. Traumatic cataracts were noted in 12 eyes in the nonpeeling group and 15 eyes in the ILM peeling group. Lensectomy or phacoemulsification along with PPV was performed during the primary procedure in 26 eyes (nonpeeling group, *n* = 12; ILM peeling group, *n* = 14). Intraocular lens placement was performed in only one eye in the ILM peeling group. During the PPV, 17 eyes (51.5%) underwent ILM peeling at the surgeon's discretion. The most common complications were vitreous hemorrhage in 25 of the 33 eyes (75.8%), retinal detachment in 22 eyes (66.7%), and endophthalmitis in 3 eyes (9%). Twenty-six of 33 patients had an IOFB, whereas 2 had an orbital foreign body. For most cases (74%), silicone oil was used for endotamponade. The time from injury to PPV was ≤7 days in 72.7% of the eyes. No intraoperative complications were identified from the case records of any surgeries. The follow-up duration ranged from 97 to 639 days. Final VA was ≥20/200 in 27 of the 33 eyes (83%), and the globe survival rate was 100%. No cases of full-thickness macular hole formation, lamellar macular hole formation, or PVR occurred during follow-up.

Table 1 provides the differences between the eyes that did and did not undergo ILM peeling. The distance from the lesion to the central fovea was significantly shorter in the ILM peeling group than in the nonpeeling group (1.94 vs. 2.75 PD, *p*=0.001). No significant between-group differences were observed in the impact site of the IOFB, rate of retinal detachment, quadrants involved, or type of endotamponade used during the surgical procedure. We found no statistically significant differences between the ILM peeling and nonpeeling groups in VA at baseline (1.68 vs. 1.58 logMAR, respectively; *p*=0.68) or at final follow-up (0.72 vs. 0.78 logMAR, respectively; *p*=0.66). The median VA significantly improved after PPV in both groups (*p* < 0.001).

**Figure 2:**
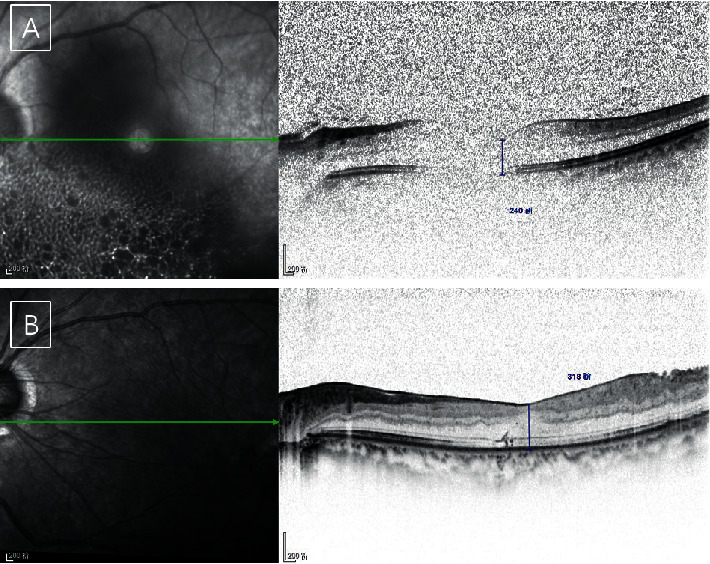
Spectral-domain optical coherence tomography scans of patients in the nonpeeling group. (a) Preoperative scan showing no epiretinal membrane. The scan is unclear because of vitreous hemorrhage. (b) A scan taken at the 35-day follow-up showing a macular pucker. A hyperreflective line at the foveal surface can also be clearly observed.

**Figure 3:**
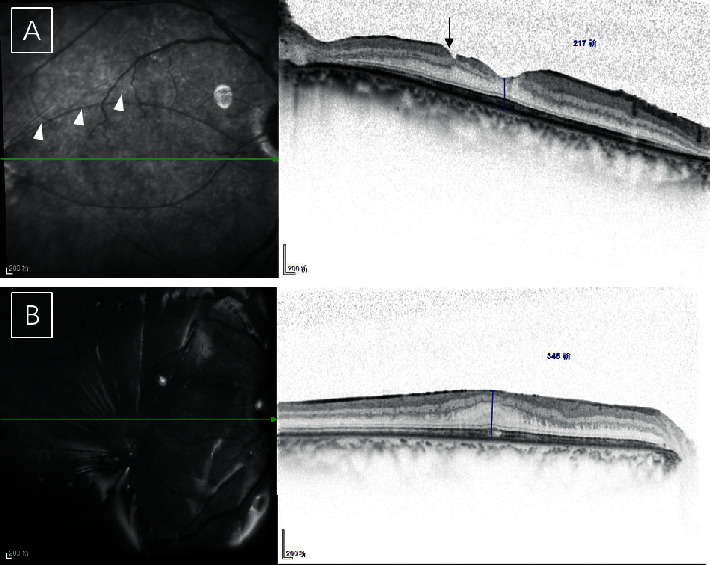
Spectral-domain optical coherence tomography scans of patients in the internal limiting membrane (ILM) peeling group. (a) The 5-month follow-up scan of a patient showing a smooth foveal surface. ILM peeling was carefully performed in the macular area and the area around the wound (white arrowheads). A pit caused by ILM peeling can be observed (black arrow). (b) Another patient developed an epiretinal membrane 3 months after the first pars plana vitrectomy.

An ERM was detected in three eyes in the ILM peeling group (17.6%), which was significantly less than that in the nonpeeling group (*n* = 8, 50.0%; *p*=0.049) (Figures 2 and 3). ERMs were also observed outside the ILM peeling region, which did not affect the fovea (Figure 3(a)). Because of the marked variability and fairly small sample size, the difference in the time to ERM between the ILM peeling group and nonpeeling group did not reach statistical significance (162.67 vs. 92.38 days, respectively; *p*=0.111). No significant difference was found in the mean foveal thickness between the ILM peeling group and nonpeeling group (207.82 vs. 259.13 *μ*m, respectively; *p*=0.402). The mean time from surgery to ERM detection was 3 months in the nonpeeling group (range, 1–6 months). Importantly, only three ERMs were detected at the mean time of 5.4 months in the ILM peeling group.

**Figure 4:**
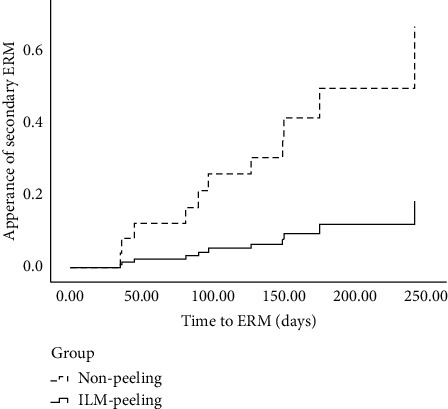
Cox proportional curves on the basis of ILM peeling. ILM, internal limiting membrane; ERM, epiretinal membrane.

Cox proportional hazards model analysis was performed in a step-by-step manner with the purpose of identifying risk factors for the development of a secondary ERM. In the multivariable models, ILM peeling (odds ratio, 0.19; 95% confidence interval, 0.04–0.91; *p*=0.04) and more severely impaired preoperative vision (odds ratio, 0.29; 95% confidence interval, 0.10–0.80; *p*=0.02) reduced the likelihood of ERM formation. The Cox proportional hazard curves show a clear difference in the proportion of patients with an ERM on the basis of ILM peeling (Figure 4).

## 4. Discussion

Several studies have shown that ILM peeling has some beneficial effects for the prevention of ERM formation in various retinal diseases, including RRD [10], proliferative diabetic retinopathy [11], and PVR [12]. For patients with an OGI who undergo a successful PPV, the formation of an ERM may be detrimental to visual function, and some may need a secondary operation. The traction of an ERM leads to image distortion, even in the eyes with poor VA. The patients can see the visual distortion because the VA does not necessarily correspond with visual quality [12]. Moreover, the peeling of a secondary ERM will still not resolve the decreased VA [13]. Secondary ERMs can adversely affect the prognosis, highlighting the importance of preventing postsurgical ERM formation in patients with OGI.

Lai et al. [14] concluded that PVD could decrease ERM formation. However, the extensive retinal folds radiating from the wound site may primarily be the result of the traction of the ILM during healing of the chorioretinal wound [15]. Therefore, it is not possible to resolve the retinal folds by simply creating a PVD; ILM peeling is also necessary.

This is the first report to describe the results of preventative ILM peeling in patients with an OGI involving the retinal area next to the macula. The results show that ILM peeling can be beneficial for decreasing the formation of a secondary ERM, resulting in morphological structures that are closer to normal than are those in patients without ILM peeling.

From a histopathologic viewpoint, the fibrotic process associated with the growth of ERMs involves diverse cell types, including hyalocytes, fibroblasts, and glial cells. In the development of an ERM secondary to OGI, the ERM is considered an early stage of PVR, which involves the posterior pole [16, 17], unlike idiopathic ERMs. The origin of a secondary ERM in OGI is likely to be RPE cells that migrate through retinal breaks toward the macular surface, on which they then proliferate [7, 17]. RPE cells may contribute to the formation of an ERM via a wound healing process [18]. In this context, the ILM is assumed to act as a scaffold for RPE cell and glial cell adhesion and proliferation, resulting in format formation of the ERM [4, 18]. For these reasons, ILM peeling can remove the ERM precursor cells from the retinal surface [19], thus preventing ERM development and even reducing the risk of posttraumatic PVR [7]. Moreover, paramacular scarring can cause irregular morphology of the macula, which induces poor visual acuity outcomes [20].

In a randomized controlled study, Kumar et al. [21] found that extramacular ERMs developed in the eyes that underwent ILM peeling in the macular area (2 PD around the fovea). Lai et al. [14] found that patients with an IOFB developed mild perifoveal retinal striae that radiated from the retinal impact sites near the fovea during postsurgical follow-up. Ren et al. [7] found that a contractile scar is formed along the surface of the impact area; the scar would then retract and wrinkle the surrounding retina. Therefore, we considered it is necessary to peel the ILM in a large area. We speculated that the development of an ERM was more likely when the retinal injury was closer to the macula. The surgeons in our study appeared more inclined to remove the ILM in patients with an injury closer to the fovea (1.94 vs. 2.75 PD). For better results, the ILM was peeled in the entire macular area (2 PD around the fovea) and the area between the fovea and the impact site, resulting in a bare region that could act as a barrier to prevent ERM occurrence (Figure 3(a)).

In patients with OGI, the presence of retinal injury, IOFBs, and vitreous hemorrhage increases the risk of ERM by inducing production of cytokines and various growth factors and promoting RPE cell dissemination [22, 23]. An observational study suggested that the average time for development of an ERM after PPV in patients with ocular trauma was 55.23 days (range, 29–72 days) [24]. In the present study, the mean time from PPV to ERM detection was longer in the ILM peeling group than in the nonpeeling group (162.67 vs. 92.38 days, respectively). However, the difference was not statistically significant because of the small sample size. Our results suggest that ILM peeling and worse preoperative VA are likely protective factors against ERM formation. There are two possible reasons for this. First, preoperative VA does not fully represent the actual degree of trauma. In this study, most patients (87.9%, *n* = 29) had severe vision loss. Preoperative VA was influenced by the presence of vitreous hemorrhage, the location and characteristics of any IOFB, and the severity of injury to the anterior segment. The second possible reason is particularly interesting: among the four patients with better preoperative VA (Snellen VA of >20/100), three developed an ERM, and they were all in the nonpeeling group. Furthermore, the average thickness of the fovea was closer to normal in the ILM peeling group. Therefore, we believe that ILM peeling deserves consideration even in patients who retain reasonable eyesight. For some patients who have poorer initial VA, ILM peeling and repair of the injury site can result in a favorable prognosis.

ILM peeling leads to mechanical trauma in the retinal microstructures, which can result in the dimple sign, pitting, temporal macular thinning, and concentric macular dark spots (Figure 3(a)) [6]. However, the effect of this anatomical damage on visual function remains controversial [4]. The patients in our ILM peeling group tended to have better postoperative VA at their last follow-up, although the trend was not statistically significant. Abdullah et al. [25] found that among patients undergoing RRD repair, those in the ILM peeling group had significantly poorer VA than those in the nonpeeling group, as well as a significant reduction in multifocal electroretinography P1-peak amplitudes. Other studies have produced similar results [26, 27]. Aras et al. [26] reported that ILM peeling during the treatment of RD did not negatively affect distance VA, which may indicate that ILM peeling has no meaningful impact on visual function in the eyes with complex lesions. In such cases, it might be more reasonable to peel the ILM.

Limitations of this study include its single-center, retrospective design, and small number of patients (*n* = 33). The constraints of a retrospective study design may lead to overestimation of the prevalence of ERM, as patients without an ERM may be excluded because of a lack of follow-up.

## 5. Conclusion

This is the first study in which visual changes and ERM development were evaluated after ILM peeling in patients with an OGI that involved areas next to the fovea. The ERM formation rate was low in the eyes with ILM peeling. This suggests that preventative treatment with ILM peeling contributed to decreasing ERM progression in patients with an OGI that involved areas next to the fovea, and such treatment did not cause a visual deficit. Furthermore, prospective randomized studies with larger sample sizes are required to confirm these observations.

## Figures and Tables

**Table 1:** Main characteristics of patients with or without ILM peeling.

Variables	ILM peeling	Nonpeeling	*P*
	(*n* = 17)	(*n* = 16)	
Mean age, years (mean ± SD)	41.65 ± 9.37	44.38 ± 10.61	0.44
Sex, *n* (%)			
Male	16 (94)	16 (100)	1
Female	1 (6)	0 (0)	
Eye, *n* (%)			
Right	10 (59)	8 (50)	0.611
Left	7 (41)	8 (50)	
Visual acuity (mean ± SD)			
Baseline logMAR	1.68 ± 0.31	1.58 ± 0.54	0.68
Final logMAR	0.72 ± 0.43	0.78 ± 0.44	0.66
Time to first PPV, days (mean ± SD)	5.82 ± 4.02	4.5 ± 5.58	0.18
Distance to fovea, PD (mean ± SD)	1.94 ± 0.70	2.75 ± 0.40	0.001
IOFB, *n* (%)			
Yes	13 (76)	13 (81)	0.737
No	4 (24)	3 (19)	
Vitreous hemorrhage, *n* (%)			
Yes	12 (71)	13 (81)	0.475
No	5 (29)	3 (19)	
Quadrants, *n* (%)			
One	6 (35)	9 (56)	0.456
Two	10 (59)	6 (38)	
Three	1 (6)	1 (6)	
Retinal detachment, *n* (%)			
Yes	10 (59)	12 (75)	0.325
No	7 (41)	4 (25)	
Endotamponade, *n* (%)			
Silicone oil	12 (71)	14 (87)	0.235
Air	5 (29)	2 (13)	
Intravitreal injection, *n* (%)			
TA	7 (41)	4 (25)	0.389
Antibiotics	0	3 (19)	
TA + antibiotics	2 (12)	1 (6)	
Ozurdex	1 (6)	1 (6)	
None	7 (41)	7 (44)	
Mean follow-up, days (mean ± SD)	262.94 ± 145.46	227.44 ± 99.67	0.533
ERM, *n* (%)	3 (18)	8 (50)	0.049

ILM, internal limiting membrane; logMAR, logarithm of the minimum angle of resolution; PD, papillary diameter; IOFB, intraocular foreign body; PPV, pars plana vitrectomy; TA, triamcinolone; ERM, epiretinal membrane.

## Data Availability

The datasets used and/or analyzed during the current study are available from the corresponding author upon request.
